# Estimation of transpulmonary driving pressure during synchronized mechanical ventilation using a single lower assist maneuver (LAM) in rabbits: a comparison to measurements made with an esophageal balloon

**DOI:** 10.1186/s13054-023-04607-2

**Published:** 2023-08-25

**Authors:** Ling Liu, Hong-Liang Li, Cong Lu, Purab Patel, Danqiong Wang, Jennifer Beck, Christer Sinderby

**Affiliations:** 1 Jiangsu Provincial Key Laboratory of Critical Care Medicine, Department of Critical Care Medicine, Zhongda Hospital, School of Medicine, Southeast University, Nanjing, 210009 China; 2 Department of Critical Care Medicine, Beijing Tiantan Hospital, Capital Medical University, Beijing, China; 3 Institute of Medical Science, University of Toronto, Toronto, Canada; 4 Department of Critical Care, St. Michael’s Hospital, Keenan Research Centre for Biomedical Science of St. Michael’s Hospital, 30 Bond Street, Toronto, ON M5B1W8 Canada; 5 Department of Critical Care Medicine, The Quzhou Affiliated Hospital of Wenzhou Medical University, Quzhou People’s Hospital, Quzhou, 324000 China; 6 Department of Pediatrics, University of Toronto, Toronto, Canada; 7 Member, Institute for Biomedical Engineering and Science Technology (iBEST) at Ryerson University and St-Michael’s Hospital, Toronto, Canada; 8 Department of Medicine and Interdepartmental Division of Critical Care Medicine, University of Toronto, Toronto, Canada

**Keywords:** Neurally adjusted ventilatory assist, Neurally triggered pressure support, Transpulmonary driving pressure, Diaphragm electrical activity, Respiratory muscle unloading, Mechanical ventilation, Lower assist maneuver, Esophageal pressure

## Abstract

**Background:**

Mechanical ventilation is applied to unload the respiratory muscles, but knowledge about transpulmonary driving pressure (Δ*P*_L_) is important to minimize lung injury. We propose a method to estimate Δ*P*_L_ during neurally synchronized assisted ventilation, with a simple intervention of lowering the assist for one breath (“lower assist maneuver”, LAM).

**Methods:**

In 24 rabbits breathing spontaneously with imposed loads, titrations of increasing assist were performed, with two neurally synchronized modes: neurally adjusted ventilatory assist (NAVA) and neurally triggered pressure support (NPS). Two single LAM breaths (not sequentially, but independently) were performed at each level of assist by acutely setting the assist to zero cm H2O (NPS) or NAVA level 0 cm H2O/uV (NAVA) for one breath. NPS and NAVA titrations were followed by titrations in controlled-modes (volume control, VC and pressure control, PC), under neuro-muscular blockade. Breaths from the NAVA/NPS titrations were matched (for flow and volume) to VC or PC. Throughout all runs, we measured diaphragm electrical activity (Edi) and esophageal pressure (*P*_ES_). We measured Δ*P*_L_ during the spontaneous modes (*P*_L__*P*_ES_) and controlled mechanical ventilation (CMV) modes (*P*_L___CMV_) with the esophageal balloon. From the LAMs, we derived an estimation of Δ*P*_L_ (“*P*_L_LAM_”) using a correction factor (ratio of volume during the LAM and volume during assist) and compared it to measured Δ*P*_L_ during passive (VC or PC) and spontaneous breathing (NAVA or NPS). A requirement for the LAM was similar Edi to the assisted breath.

**Results:**

All animals successfully underwent titrations and LAMs for NPS/NAVA. One thousand seven-hundred ninety-two (1792) breaths were matched to passive ventilation titrations (matched Vt, *r* = 0.99). *P*_L_LAM_ demonstrated strong correlation with *P*_L___CMV_ (*r* = 0.83), and *P*_L__*P*_ES_ (*r* = 0.77). Bland–Altman analysis revealed little difference between the predicted *P*_L___LAM_ and measured *P*_L___CMV_ (Bias = 0.49 cm H2O and 1.96SD = 3.09 cm H2O). For *P*_L__*P*_ES_, the bias was 2.2 cm H2O and 1.96SD was 3.4 cm H2O. Analysis of Edi and *P*_ES_ at peak Edi showed progressively increasing uncoupling with increasing assist.

**Conclusion:**

During synchronized mechanical ventilation, a LAM breath allows for estimations of transpulmonary driving pressure, without measuring *P*_ES_, and follows a mathematical transfer function to describe respiratory muscle unloading during synchronized assist.

**Supplementary Information:**

The online version contains supplementary material available at 10.1186/s13054-023-04607-2.

## Introduction

Mechanical ventilation is essential for the treatment of respiratory failure and can be applied either by fully controlling ventilation in patients who are passive and not breathing (CMV), or by providing partial ventilatory assist, where the patient breathes spontaneously in conjunction with the ventilator. Partial ventilator assist can be synchronized to the diaphragm electrical activity (Edi), either with assist delivered proportionally (as during neurally adjusted ventilatory assist, NAVA) [1] or with a fixed level of pressure (as with neurally triggered and cycled pressure support, NPS) [2, 3].

During CMV, the ventilator overcomes the total respiratory system load (resistive and elastic) on its own, and therefore, the pressure required to inflate the respiratory system (*P*_RS_) to a given volume is known, according to the equation of motion. During CMV, the respiratory muscles do not play any role in generating the pressure–volume characteristics of the respiratory system. By measuring the esophageal pressure (*P*_ES_) during passive ventilation, which provides a surrogate for the chest wall recoil pressure (*P*_CW_), the transpulmonary pressure (*P*_L_) during CMV can be obtained (*P*_L_CMV_ = *P*_RS_ − *P*_CW_).

However, during assisted ventilation, when the patient breathes spontaneously, new forces contributing to *P*_L_ come into play. During synchronized assist, the patient’s forces, generated by the respiratory muscles, add to the forces of the ventilator, overcoming the respiratory load together. The relative contribution of the patient and the ventilator to overcoming the load will change with the level of assist. We have previously described the fraction of patient volume to tidal volume during NAVA, in both animals and humans [4–6]. The so-called “patient-ventilator breath contribution (PVBC) index”, calculated as: volume of unassisted breath divided by the volume of the assisted breath (for a given Edi), was related to the patient’s contribution to transpulmonary pressure (*P*_ES_/*P*_L_), and hence an index of unloading. Importantly, PVBC needed to be multiplied by-itself (i.e., squared) to improve and linearize the relationship with *P*_ES_/*P*_L_ [4, 5].

During synchronized assist, there are several knowns and unknowns: From the ventilator display, we know the tidal (total) volume, but we do not know the patient’s volume alone, nor the ventilator’s volume. We can measure the patient volume alone by acutely lowering the assist, “lower assist maneuver” (LAM), for one single breath. This can be achieved by pre-programming the ventilator to lower the assist, for one single breath, at timed intervals decided by the user. Whether or not this LAM volume is the same volume the patient can generate (for the same Edi) during an assisted breath (when flow is higher) is not known. Regarding pressure, ventilator pressure delivered is displayed (*P*_VENT_), but we do not know the patient’s pressure, and hence, the tidal inspiratory driving transpulmonary pressure (Δ*P*_L_) is unknown (unless an esophageal balloon is in place).

The primary aim of the present study was to evaluate an estimate of transpulmonary driving pressure (Δ*P*_L_) based on the volumes generated during the LAM breaths and the assisted breaths. A secondary aim was to determine the underlying principles of unloading and to identify the contributions of the respiratory muscles and the ventilator during synchronized assist, in terms of pressure and volume (to solve the unknowns).

## Methods

The study was approved by St. Michael’s Hospital Animal Care and Use Committee (ACC 482). Care and handling of the animals were performed according to the Canadian Council on Animal Care.

### General protocol

In rabbits, titrations of increasing assist levels, with two neurally synchronized modes of assist: NAVA and NPS (studied independently) were performed. LAM breaths were introduced for both modes, at each level of assist. Four separate conditions were tested: no load, low or high resistive loading and chest wall banding. This was followed by a period of CMV under neuromuscular blockade (using the equation of motion with matching flow and volume); we also measured esophageal pressure throughout the study to add to the validation of the surrogate measure of Δ*P*_L_.

#### Animals and instrumentation

Twenty-four adult male New Zealand white rabbits (Charles River Labs, St Constant, Quebec, Canada) with a mean body weight of 2.9 kg (range 2.7–3.2) were studied. Prior to instrumentation, animals were initially anaesthetized by an intramuscular bolus of ketamine hydrochloride (35 mg/kg) and xylazine (10 mg/kg), followed by continuous intravenous infusion of ketamine hydrochloride (10 ml/kg per hour), xylazine (2 mg/kg per hour).

During the entire protocol, Lactated Ringer’s solution (5 mL/kg per hour) was continuously infused intravenously with an infusion pump. Arterial blood pressure (Pd 23, Gould Inc. Cleveland, OH) and arterial blood gas measurements (RADIOMETER ABL800 FLEX, Mississauga, Canada) were obtained from an ear artery with an indwelling arterial line. Transcutaneous oxygen saturation was monitored with pulse oximetry at the tail (NONIN 8600 VTM, Nonin Medical Inc., Plymouth, MN). Edi was measured with an array of small sensors placed on an 8F oro-gastric catheter, with a balloon mounted for measurement of esophageal pressure (*P*_ES_) and gastric balloon for gastric pressure (*P*_GA_) (Neurovent Research Inc. Toronto, Canada). Proper positioning of the catheter was confirmed using a dedicated window on a Servo-i ventilator (Maquet, Sweden), as well as inspiratory occlusion maneuvers, repeated throughout the study, when spontaneous breathing was present. A tracheotomy was performed, and an endotracheal tube (size 4.0) was inserted. An additional physical (instrumental) dead space was inserted into the circuit to slightly increase respiratory drive and kept in place for the entire study. The Servo-i ventilator was used and connected via the tracheostomy. Ventilator pressure was measured at the y-piece (*P*_VENT_). Flow was measured via a Hans Rudolph (Model 4500) pneumotach connected at the y-piece as well.

The following waveforms were measured and recorded continuously throughout the protocol: Edi, *P*_ES_, *P*_GA_, *P*_VENT_, flow. Regarding vital signs, we monitored blood pressure (BP), heart rate (HR), and oxygen saturation (SAT). Volume was obtained by integrating the flow signal.

#### Modes, titrations, and maneuvers:

The protocol consisted of four main steps: (1) titration of spontaneous breathing modes (NAVA then NPS, or vice versa), (2) neuromuscular blockade, (3) pressure control titration, and (4) volume control titration.

*NAVA mode*: Using the Servo-i ventilator, the assist is triggered when the Edi reaches a threshold deflection from baseline, and pressure is delivered in proportion to Edi times the NAVA level (cm H2O/uV). The breath is cycled off when Edi decreases to 70% of its peak. A fixed, user-defined PEEP is applied during exhalation (zero cm H2O in the present study). For the titrations, the NAVA level was increased every 6–8 min in steps of 0.3, starting at NAVA level 0.6 up to 2.4 cm H2O/uV.

*NPS*: Also using the Servo-i ventilator, we used a newly implemented neurally triggered pressure support, a mode delivering fixed level of targeted pressure, triggered and cycled off by Edi, with the same trigger and cycling criteria as in NAVA. NPS was increased every 6–8 min in steps of 2 cm H2O (starting at 4 up to 14 cm H2O). PEEP applied during exhalation was also zero cm H2O.

**Fig. 1:**
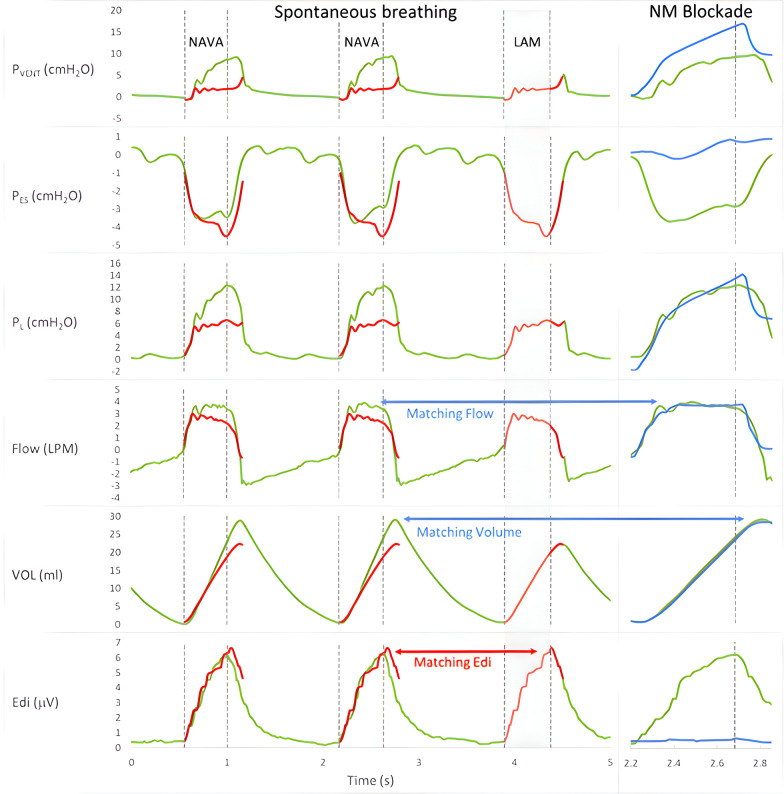
Sample waveforms during neurally adjusted ventilatory assist (NAVA) and volume control (VC) to demonstrate protocol steps and analysis. Signals were obtained in one animal breathing spontaneously in the NAVA mode (first three breaths), or passively ventilated in VC mode (far right), both shown with resistive load, NAVA level 1.5 cm H2O/uV. From top to bottom, single breath waveforms are presented for ventilator pressure (*P*_VENT_), esophageal pressure (*P*_ES_), transpulmonary pressure (*P*_L_), flow and volume (VOL), and electrical activity of the diaphragm (Edi). The first three breaths (left to right) are NAVA breaths during spontaneous breathing. The third breath is a low assist maneuver (LAM), shown in red. The respective waveforms from the LAM (red) were superimposed on the NAVA breaths (green) to demonstrate matching of Edi values. The fourth breath, on the far right, was taken during neuromuscular (NM) blockade, so the subject is not activating their respiratory muscles (Edi is flat) and ventilation is passive (*P*_ES_ goes positive for the breath) during Volume Control mode (blue lines). We superimposed the assisted breath with the CMV breath to demonstrate the matching of volume and flow for spontaneous (green) and passive (blue) breaths

*Lower assist maneuver (LAM)*: During both NAVA and NPS, two LAM maneuvers were performed independently (but not sequentially) during the 6–8 min periods. This was achieved automatically by pre-programmed lowering of the NAVA level to 0 cm H2O/uV, or NPS to 0 cm H2O. Note that the Servo-i ventilator will always provide a minimum pressure of 2 cm H2O during the LAM (see Fig. 1, red waveforms) in both NAVA and NPS modes.

Occlusions and end-inspiratory holds were also performed manually, once, at each level of assist, as routine maneuvers in this experimental model.

Following the NAVA and NPS titrations, neuro-muscular blockade was induced in order to obtain passive mechanical ventilation, and to obtain breaths for matching the spontaneous breathing titrations. Neuromuscular blockade was achieved by administering 0.3 ml of a solution of succinyl choline or pancuronium (0.1 ml of drug, 0.9 ml saline) until Edi was absent (~ 0 uV) (indicating passive ventilation, see Fig. 1, flat blue line in bottom right panel).

*Pressure control (PC) mode* and *volume control mode (VC)* were used in order to “match” breaths of similar volumes/flow as obtained during the NAVA or NPS runs (“matching” refers to later, off-line analysis, see below “Off-line analysis”). PC was increased every 20–30 s in steps of 1 cmH2O, from 6 to 24 cmH2O. VC was increased every 20–30 s, and respiratory rate and duty cycle (Ti/Ttot) ratio were varied to get approximately 0.25–0.3 LPM steps, from 3 to 12 LPM.

During passive ventilation, we also measured the volume generated for 2 cm H2O (in PC mode) in order to correct for the 2 cm H2O during the LAM.

#### Respiratory loads

The above-mentioned steps in the protocol were repeated with different respiratory loads:

*Resistive load*: Animal was breathing through a resistor inserted at the endo-tracheal tube. More specifically, the increased resistances were achieved by inserting a plug with holes to narrow the diameter of endotracheal tube. Two different resistors were evaluated (“Low” and “High” resistors were quantified as 95 cm H2O/l/s at a flow rate of 50 ml/s and 156 cm H2O/l/s at a flow of 50 l/s, respectively).

*Banding*: An athletic bandage was wrapped from the lower rib cage to the lower abdomen, with the aim of increasing the baseline of the *P*_GA_ waveform by 5–6 cmH2O.

Animals were ethically euthanized at the end of the study, according to ACC guidelines.

### Off-line analysis

#### Manual and automated selection of assisted breaths and LAM breaths (inclusion criteria) obtained during NAVA and NPS

Both manual and automated analysis was performed, the latter to reduce selection/data bias.

During the manual analysis, breath-by-breath selection was first performed by toggling through all the assisted breaths (NPS and NAVA) and the LAM breaths, where we displayed simultaneously on a computer monitor: Edi, *P*_VENT_, flow, and volume. Cursors were placed at the beginning of the Edi increase (dashed vertical lines, Fig. 1), and the peak of Edi (solid vertical lines, Fig. 1). Cursors were placed for the beginning and end of the inspiratory flow and *P*_VENT_ curves as well (for simplicity, not shown in Fig. 1).

For the automated analysis, the waveform time-points for the assisted breaths and the LAM breaths were detected automatically: Edi onset and Edi peak were obtained by taking the “state” of the ventilator (a digital signal collected from the Servo-I ventilator). Hence, the onset of ventilator pressure and inspiratory flow were also automatically detected and stored. This is possible because the neural (Edi) signal is the command for triggering and cycling-off both modes.

*Matching assisted breaths with LAM breaths using Edi inclusion criteria*: For both manual and automated analysis, inclusion criteria for Edi-matched breaths (assisted breaths compared to LAM) were adopted (also shown as green and red waveforms in Fig. 1): Edi peak > 1.5 uV; neural inspiratory time > 200 ms. A priority requirement for inclusion into the analysis was that the Edi waveform (start to peak) needed to “match” by an arbitrary value of at least *R*^2^ = 0.85 (determination coefficient calculated with regression analysis of Edi during assist, and Edi during LAM).

Note that the breath variables in the present analysis are analyzed from start to peak of Edi, unless otherwise mentioned.

*Matching spontaneous assisted breaths with passive mechanical ventilation breaths*: The assisted breaths that had a “Edi-matched” LAM were then stored for later comparison of the breaths obtained during the PC or VC mode. Volume and flow waveforms for single breaths from NAVA/NPS were simultaneously displayed on top of those obtained during PC and VC (similarly to that shown in Fig. 1, right side green waveform superimposed on blue waveform), and when matched for the flow and volume profiles, were tagged as “matched”. This matching of spontaneous and passive breaths was carried out initially by a group of 4 investigators (CS, LL, JB, NC) observing the single breath waveforms on a large computer screen, followed by verification of individual investigator analysis (CS, LL, PP).

#### Calculated and predicted respiratory pressures

During assisted ventilation (NPS/NAVA), Δ*P*_L_ was calculated as *P*_VENT_–*P*_ES_, obtained at the nadir of *P*_ES_, and will be referred to as *P*_L__*P*_ES_.

During CMV, Δ*P*_L_ was calculated as *P*_VENT_–*P*_CW,_ the latter obtained from the esophageal balloon during passive ventilation and will be referred to as *P*_L___CMV_.

Total respiratory system pressure (*P*_RS_) was taken during passive ventilation as *P*_VENT_.

Note that when *P*_ES_ is reported, Δ*P*_ES_ was used (i.e., swing in esophageal pressure from baseline to nadir).

All other variables were taken at peak Edi.

The predicted Δ*P*_L_—based on the LAM—was calculated as described below.

The known variables at the start were:

(I) *LAM volume* (i.e., patient-generated volume alone, during a single breath where the assist is reduced to 0 cm H2O in NPS, and 0 cm H2O/uV during NAVA).
(II) *Tidal volume* (total volume delivered and includes both patient and ventilator) during NPS or NAVA.
(III) *Ventilator pressure* during NPS or NAVA.

##### We needed to solve

*Ventilator volume* (VOL_VENT_) alone.

This would allow us to obtain the global load the ventilator (or the patient, or both) needs to overcome, so need to calculate *P*_VENT_/VOL_VENT_.

1. $${\text{Ventilator}}\;{\text{Volume}}\;({\text{VOLVENT}}) = {\text{Tidal}}\;{\text{volume}}{-}({\text{LAM}}\;{\text{volume}}*{\text{PVBC}})$$,

Where PVBC = (LAM volume/Tidal volume)

2. Calculate load factor: *P*_VENT_/VOL_VENT_

3. Calculate *P*_L___LAM_ = Tidal volume * load factor

*P*_L___LAM_ is the driving pressure that BOTH patient and ventilator need to generate to overcome the global load. *Note that we also estimated PL without the PVBC correction (see Additional file 1).

##### *P*_L___LAM_ corrections for pressure of 2 cm H2O during LAM

Since 2 cm H2O is applied during the LAM, corrections were applied:

1. 2 cm H2O is subtracted from the ventilator pressure (“pressure correction”).
2. During neuro-muscular paralysis (passive ventilation), PC mode of 2 cm H2O was delivered to see the resulting volume that was obtained. Thereafter, the volume during LAM was corrected for this volume (which was 3 or 4 ml on average) (“volume correction”).

### Statistical analysis

Statistical analysis was performed using Sigma Stat (v.10). Bland–Altman plots were used for comparison of measured and estimated pressures. Regression analysis was used to correlate Edi and Δ*P*_ES_.

## Results

Twenty-four animals underwent all steps of the protocol with no adverse side effects to report.

Figure 1 demonstrates single breath waveforms recorded in one animal during the NAVA mode (spontaneous breathing) and shows the ventilator-assisted breath (green) as well as the LAM (red). Tracings are obtained at a NAVA level of 1.5 cm H2O/uV with the low resistance in place. For matching of spontaneous breaths and LAM: During manual analysis, 8063 “assist-to-LAM” matches were included; during automated analysis, 52,287 matches were found.

Passive ventilation (VC mode after NM blockade) tracings are displayed to the right (blue) and demonstrate superimposed volume and flow curves (“Matching” of spontaneous (green) and passive (blue) breaths), shown in Fig. 1. Tidal volumes correlated nearly perfectly for the matched assisted and CMV breaths (*r* = 0.99).

Examples of waveforms in one representative subject during NPS with increasing levels of assist are presented in the Additional file 2, see Figure E1.

**Fig. 2:**
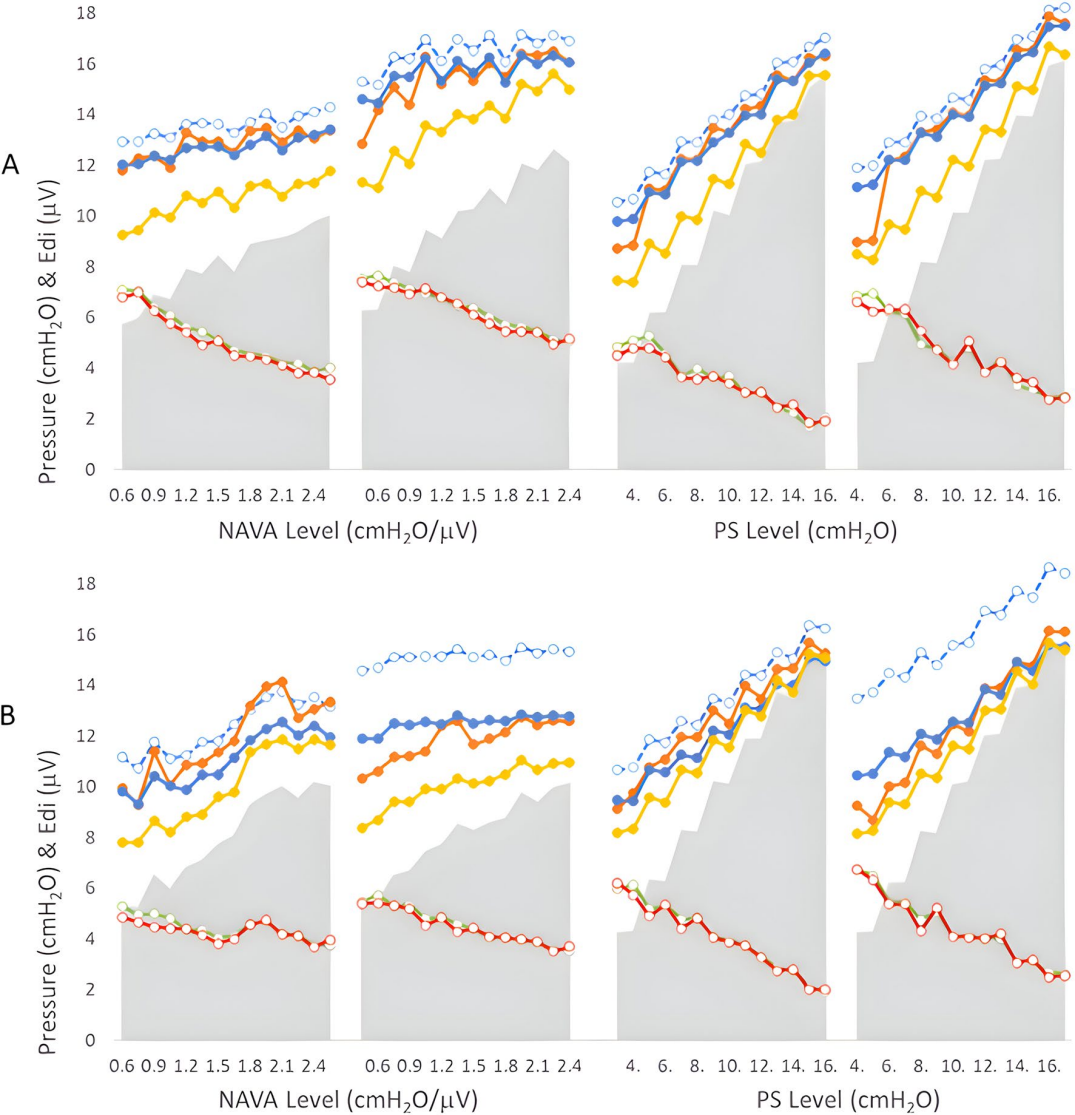
Group mean data for the measured and predicted pressures during all conditions. Measured and predicted pressure, as well as diaphragm activity (*y* axis), are provided for increasing assist (*x* axis) with NAVA (left panels), and NPS (right panels), as indicated. The results for resistive loads (low and high) PANEL A and unbanded and banded PANEL B conditions are presented. *P*_VENT_ is displayed as the pale grey solid shading; *P*_RS_, blue empty circles and blue dashed lines; *P*_L___LAM_ orange, solid circles; *P*_L___CMV_ blue solid circles blue solid line; *P*_L__*P*_ES_, yellow solid circles and solid line; Note that Edi assisted (green circles) and Edi LAM (red circles) are nearly identical

Figure 2 demonstrates group mean data for the measured pressures and *P*_L___LAM_ as well as diaphragm activity (*y* axis) during increasing assist (*x* axis) with NAVA (left panels), and NPS (right panels), as indicated. The results for resistive loads (low and high) and unbanded and banded conditions are presented in the top and bottom panels, respectively.

As expected, with all loads, stepwise increases in the NAVA level increased *P*_VENT_, as do the pressures with increasing NPS (*P*_VENT_ is shown as pale grey shaded area for emphasis). The overall response to the increasing assist levels was a gradual reduction in Edi for both the assisted (green circles) and the LAM (red circles) values (average decrease in Edi ranged from 30% to more than 50% from lowest to highest assist).

The predicted pressure *P*_L___LAM_ (closed orange circles, orange line), was closely similar to *P*_L___CMV_ for resistive loads during both NAVA and NPS. During unbanded conditions, *P*_L___LAM_ was also similar to *P*_RS_, and slightly higher than *P*_L___CMV_ (by a few cm H2O), but this phenomenon changed when banding was applied. Banding increased *P*_RS_ obtained during CMV (blue dashed line). With banding, *P*_L___LAM_ was similar to *P*_L___CMV_ and was far below the measured *P*_RS_.

The results for another estimate of *P*_L_ (which we termed “Pα”), where the volume of the LAM is not corrected for PVBC, is presented in the Additional file 3, see Figure E2. Pα clearly overestimated all *P*_L_ measures and responded unexpectedly with increasing levels of assist during the titrations.

**Fig. 3:**
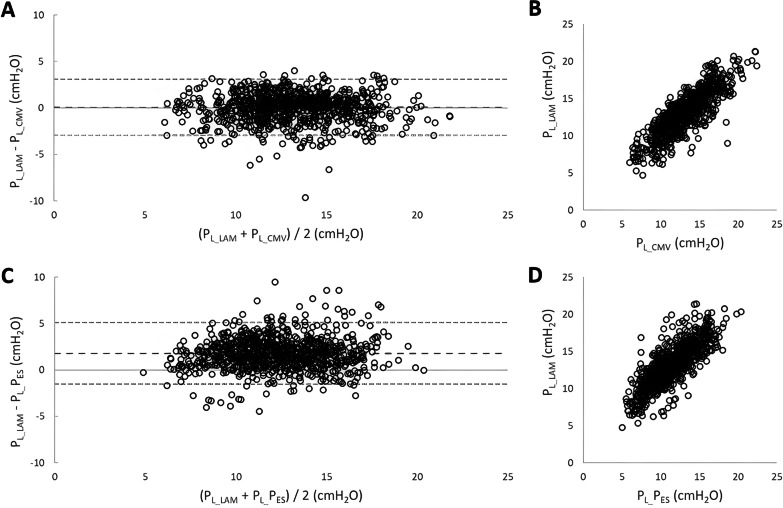
Bland–Altman plots and regression analysis for comparisons between *P*_L___LAM_ and *P*_L___CMV_, and *P*_L___LAM_ and *P*_L__*P*_ES_. Panels **A** and **C** (top graphs): Left: difference of predicted *P*_L___LAM_ and measured *P*_L___CMV_ (*y* axis) versus the mean of predicted *P*_L___LAM_ and measured *P*_L___CMV_ (*x* axis). Right: regression analysis between predicted *P*_L___LAM_ (*y* axis) and measured *P*_L___CMV_ (*x* axis), *y* = 0.8491*x* + 2.0646, *R*2 = 0.7213. Panels **C** and **D** (bottom graphs): Left: difference of predicted *P*_L___LAM_ and measured *P*_L__*P*_ES_ (*y* axis) versus the mean of predicted *P*_L___LAM_ and measured *P*_L__*P*_ES_ (*x* axis). Right: regression analysis between predicted *P*_L___LAM_ (*y* axis) and measured *P*_L__ *P*_ES_ (*x* axis), *y* = 0.8413*x* + 3.6023, *R*2 = 0.6544

Figure 3 shows Bland–Altman plots (left panels A and C) and regression analysis (right panels B and D) for comparisons between the predicted *P*_L___LAM_ and measured *P*_L___CMV_ (Panel A), as well as predicted *P*_L___LAM_ and measured *P*_L__*P*_ES_ (obtained during spontaneous breathing modes), Panel C. The analysis includes the data from all animals, all assist levels, and all conditions (*n* = 1792). In general, *P*_L___LAM_ agreed very well with both *P*_L___CMV_ and *P*_L__*P*_ES_. Linear regression analysis demonstrated strong correlation between *P*_L___CMV_ and *P*_L___LAM_ (*r* = 0.83) Panel B, and *P*_L__*P*_ES_ and *P*_L___LAM_ (*r* = 0.77) Panel D. Bland–Altman analysis revealed very little difference between the predicted *P*_L___LAM_ and measured *P*_L___CMV_ (Bias of 0.49 cm H2O and a 1.96SD of 3.09 cm H2O). For *P*_L__*P*_ES_, the Bias was 2.2 cm H2O and the 1.96SD was 3.4 cm H2O.

We decided to investigate the variability of the error of *P*_L___LAM_ when it was compared to *P*_L_CMV_: Frequency distribution analysis of all the data indicated that 50% of *P*_L___LAM_ values were similar to *P*_L___CMV_ with an error < 1 cm H2O. 75–80% of the values fell within 1–2 cm H2O error around the bias, and 94% of all values were off by < 3 cm H2O.

**Fig. 4:**
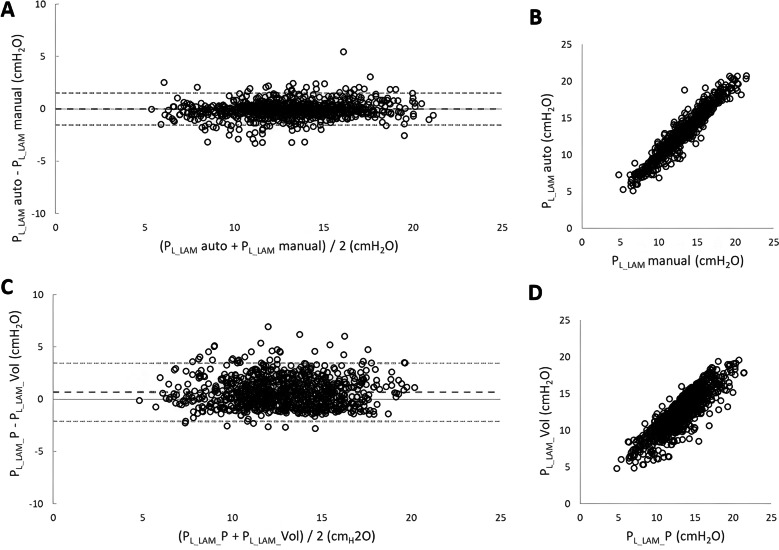
Bland–Altman plots and regression analysis for comparisons between *P*_L___LAM_ with manual versus automated analysis, and *P*_L___LAM_ with volume correction versus *P*_L___LAM_ with pressure correction. **A** and **B** (top graphs): Left: difference of *P*_L___LAM_ manual and *P*_L___LAM_ automated versus the mean of *P*_L___LAM_ manual (*x* axis) and *P*_L___LAM_ automated (*y* axis). Right: regression analysis between *P*_L___LAM_ manual (*x* axis) and *P*_L___LAM_ automated (*y* axis), *y* = 1.0031*x* + 0.0543, *R*2 = 0.9276. **C** and **D** (bottom graphs): Left: difference of *P*_L___LAM_ “volume correction” and *P*_L___LAM_ “pressure correction” (*y* axis) versus the mean of *P*_L___LAM_ “volume correction” and *P*_L___LAM_ “pressure correction” (*x* axis). Right: regression analysis between *P*_L___LAM_ “pressure correction” (*x* axis) and *P*_L___LAM_ “volume correction” (*y* axis), *y* = 0.8694*x* + 1.0407, *R*2 = 0.7584

Figure 4 shows a Bland–Altman plot (left panel, Panel A) and regression analysis (right panel, Panel B) for comparisons of mean values of *P*_L___LAM_ obtained during manual analysis and *P*_L___LAM_ obtained during automated analysis (Panel A). An excellent correlation was found between the two methods (*r* = 0.96, Panel B), and the bias was 0.014, and 1.96SD 1.5 cm H2O.

Bland–Altman analysis of *P*_L___LAM_ with “pressure correction” versus *P*_L___LAM_ with “volume correction” (Panel C) also revealed good correlation (*r* = 0.87, Panel D) with a bias 0.7, and 1.96SD 2.8 cm H2O.

**Fig. 5:**
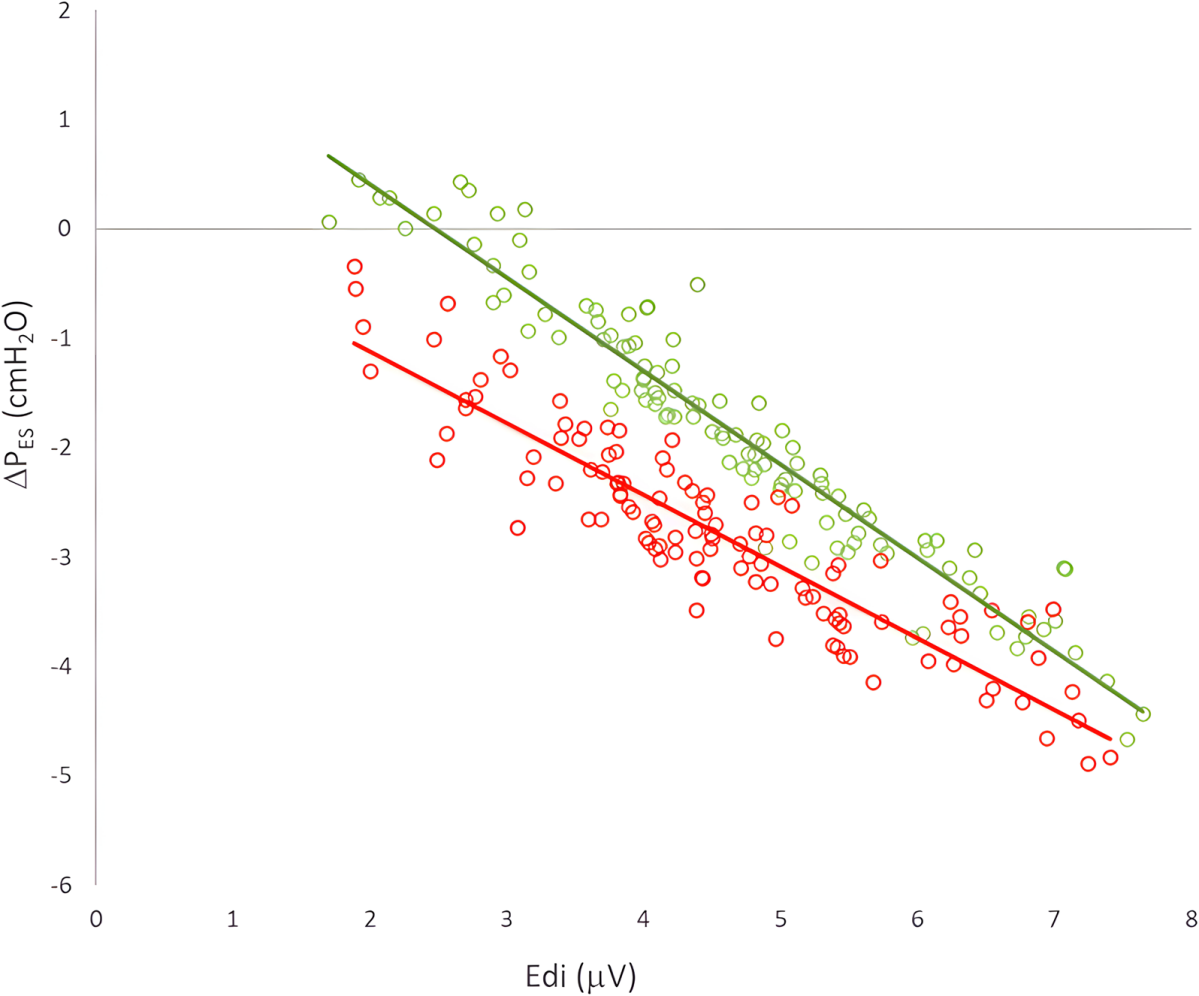
Relationship between Edi and Δ*P*_ES_ for assisted and LAM breaths. *Y* axis: Δ*P*_ES_; *x* axis: Edi peak. Both are plotted for the assisted breaths (green symbols) and LAM (red symbols). Data are presented for all animals, in all conditions and in both NAVA and NPS. Regression formulas are: for assisted (green line) *y* = − 0.8554*x* + 2.1273; *R*2 = 0.9214. For LAM (red line), *y* = − 0.6561*x* + 0.1938; *R*2 = 0.8387

Figure 5 shows the relationship between Edi (*x* axis) and *P*_ES_ (taken at Peak Edi, *y* axis) for the assisted breaths (green symbols) and LAM breaths (red symbols). Data are presented for all animals, in all conditions and in both NAVA and NPS. Note the difference in slopes and intercepts for the LAM and the assisted data. Whereas *P*_ES_ for the LAM was negative for the lowest Edi values, *P*_ES_ for the assisted breaths reached positive values when Edi was at its lowest (i.e., at highest levels of assist). The correlation values for assisted Edi versus *P*_ES_ were *r* = 0.96, and for the unassisted Edi versus *P*_ES_, *r* = 0.92. The figure shows a clear “uncoupling” between Edi and *P*_ES_ during the assisted breaths, the uncoupling being more prominent as assist increases (right to left as Edi goes down).

## Discussion

### Summary of findings

The present study introduces a new estimation for transpulmonary driving pressure during neurally synchronized assisted ventilation. A simple maneuver is required: an acute lowering of assist from the current setting to zero, for one single breath. We refer to this as the lower assist maneuver (LAM), for which there is one important requirement, similar neural drive (Edi) for the LAM and the assisted breath (an example is shown in Fig. 1). Our physiological measurements allowed us to describe a mathematical equation, to estimate Δ*P*_L_ “*P*_L___LAM_”, without the use of an esophageal balloon. Our study also examines the principles of unloading during synchronized, assisted mechanical ventilation. We demonstrate in animals with elastic or resistive loads, that increasing assist with either NAVA or NPS, unloads the breathing by a combined action of volume supplementation and load subtraction.

### *P*_L___LAM_ and simplified derivation

By re-arranging the equation for *P*_L___LAM_ provided in the Methods section, we obtain a final simpler derivation for this estimate, only requiring measurements for the current ventilator setting, and the LAM (See Additional file 1 for details of derivation):

$${\text{P}}_{{{\text{L}}\_{\text{LAM}}}} = \Delta {\text{P}}_{{{\text{VENT}}}} /1 - ({\text{PVBC}}^{2} )$$

where Δ*P*_VENT_ = (*P*_VENT_ during assist − *P*_VENT_ LAM) and

PVBC = LAM Volume/Tidal Volume during assist.

In other words, *P*_L___LAM_ is essentially a measurement of the ventilator pressure difference between two assist levels, corrected for a fraction of the patient’s contribution to tidal volume.

With the elastic load (banding), *P*_L___LAM_ did not “see” (measure) the extra-pulmonary load, in either NAVA nor in NPS. These results collectively suggest that *P*_L___LAM_ is actually predicting closer to *P*_L_, and not *P*_RS_ since it did not “sense” the increase *P*_CW_.

### Assumptions

Three assumptions are important to remember in the interpretation of our results and the below discussion:

(i) The neural respiratory drive, or Edi, represents the recruitment and firing rate of activated motor units. Even though each muscle fiber within a motor unit has its own defined and fixed crossbridge cycling rate, the Edi will reflect an average of the shortening velocities for all fibers within the bundle [7, 8]. Therefore, two breaths with equal peak Edi have the same average motor unit activation and velocity of shortening. A breath with an increased Edi has an increased velocity of shortening (as shown with sonomicrometers [9] and a breath with a lower Edi will have a lower velocity of shortening.
(ii) The esophageal pressure in the present study is not used in the final estimate of *P*_L___LAM_. Rather it is used for three reasons:
  1. to obtain the chest wall recoil pressure during paralysis, allowing transpulmonary driving pressure to be calculated (as *P*_RS_ − *P*_CW_) during passive ventilation.
  2. to describe the transpulmonary driving pressure during spontaneous breathing and assisted ventilation (*P*_VENT_ − *P*_ES_)
  3. To relate Edi and *P*_ES_ as an efficiency measurement of the subject.
(iii) During paralyzed conditions, the pressure required to distend the lung to a given volume follows the equation of motion. We obtained flow and volume measurements during spontaneous breathing (NAVA or NPS) and matched them to the same waveforms as during paralysis and passive CMV.

### *P*_L___LAM_ interpretation

What does *P*_L___LAM_ measure? Our observations that *P*_L___LAM_ did not recognize the increased elastic load (*P*_L___LAM_ not related to *P*_RS_ with banding) led us to conclude that the chest wall does not influence *P*_L___LAM_. In fact, STEP 3 in the derivation of *P*_L___LAM_ only involves *P*_VENT_ and VOL_VENT_ (recall the patient’s volume is subtracted). This provided us with a “patient effort-independent” load factor. This reflects the pressure required by the ventilator to achieve the ventilator’s volume. During passive ventilation, we subtracted the chest wall recoil pressure from *P*_RS_ to obtain the lung-distending driving pressure, *P*_L___CMV_. This could be interpreted as “pure”, passive lung inflation by the ventilator. *P*_L___LAM_ measured very closely to *P*_L___CMV_, as revealed by the Bland–Altman and regression analysis, using several different levels of assist, different loads, and two modes of synchronized ventilation with different pressure profiles (Fig. 3, Panel A). *P*_L___LAM_, therefore, likely reflects the pressure required to inflate the lung to the volume up to peak Edi. In passively ventilated rats, in an open-chest model (chest wall load removed), there was excellent agreement between transpulmonary pressures and alveolar pressure computed using capsule pressures [10]. Perhaps *P*_L___LAM_ reflects alveolar pressure, providing a new possibility for this measurement in spontaneously breathing, mechanically ventilated subjects.

What are the limits of the transfer function? This depends on the PVBC. If the patient’s respiratory drive is barely present, there would be little patient contribution (low PVBC) as well as low volume during the LAM maneuver. In effect, this situation could be thought of as “approaching passive ventilation”, and therefore, *P*_L___LAM_ would approach *P*_VENT_ since the denominator of the equation is close to unity (i.e., 1). This means we would start to measure the *P*_CW_. In other words, when the patient is not contributing, the muscles generate no pressure, which means no pressure to expand the chest wall, and the ventilator does all the work. If volume during the LAM > 0 ml, then there is at least some patient pressure sufficient to generate some volume, and the muscles are being used to expand the chest wall. At the other extreme, if the patient is doing all the work (and the ventilator barely anything), then PVBC would be close to 1, bringing the denominator of the equation close to 0, a mathematical uncertainty. However, it is nearly impossible to have a PVBC ~ 1, it would imply no ventilator assist at all. The highest PVBC we observed in the present study was ~ 0.8.

### Principles of unloading during NPS and NAVA

In the present study, we performed stepwise increases in assist (increased NAVA levels, or increased NPS levels) as the main intervention. The physiological response to increasing NAVA levels (not the acute changes but the changes seen after several breaths) has been described in both animals and critically ill patients [11, 12]. For every increase in assist, the response could be a maintained Edi, with an observed increase in ventilator pressure, flow and volume, as demonstrated in one subject in Figure E1 (Additional file 2) from 4 to 8 cm H2O. Or instead, increasing the assist could result in down-regulation of Edi with the ventilator pressure, flow and volume staying the same. Another response could be somewhere in between, all depending on the respiratory demand. At highest levels of assist, the response is usually a down-regulation of Edi to prevent overdistension, according to the vagally mediated HB inflation reflex or because of improved ventilation. Note that even at highest levels of assist (during neurally controlled modes), the Edi may be down-regulated as low as 40% of its highest value, but never eliminated [11, 12]. In other words, there will always be some neural drive even at very high levels of assist in neurally controlled modes of mechanical ventilation. Note, this is different from the mechanical unloading (*P*_ES_), which could be reduced (more positive values)/eliminated because of uncoupling.

### LAM versus assisted breathing

During acute changes in assist, whether higher or lower, for one single breath, it has been shown that the Edi can remain the same (same peak value and same time to peak) “one breath to the next” [4, 5, 13]. Viale [13] was the first to demonstrate the immediate unloading between 2 sequential breaths, one without assist, and one with assist, during BIPAP ventilation in patients. The Edi and Pdi were measured during the alternating breaths (assist, no assist). The Edi values were similar from one breath to the next, however the Pdi was reduced for the assisted breaths. This was found for both animals [4] and humans [5] in our previous work.

Going to a sudden increase in assist, (NAVA level or NPS), since the Edi peak remains the same, the velocity of activation/shortening of the diaphragm is the same. When the ventilator provides more assist and more volume in this same time period (i.e., volume supplementation) it results in an increased velocity of air delivery compared to when there was less assist. Hence, the velocity of shortening of the diaphragm is overtaken by the ventilator, thereby removing the load against which the diaphragm can contract (load subtraction).

The relative imbalance of flow delivery, faster than the diaphragm activation/shortening velocity, causes the diaphragm to lose its force-generating ability, as described in single fiber experiments with negative loads [14]. Therefore, for a given Edi (peak and time to peak), the diaphragm is “uncoupled” and loses efficiency, as reflected by the reduced swings in *P*_ES_ that we observed (Fig. 5 and Figure E1 in Additional file 2, green dashed line). The flow increase (with increasing assist) not only expands the lung (volume supplementation) but also subtracts a fraction of the load that the diaphragm can contract against. As you increase assist, the increase in flow (velocity of air delivery) is more and more dominant by the ventilator, perhaps until very high assist levels, when the subject can no longer generate flow, (despite Edi is present), but the ventilator performs all the lung distending pressure. This has previously been demonstrated in healthy subjects, breathing through a mouthpiece with increasing NAVA levels: Both during quiet breathing and during TLC maneuvers, the transdiaphragmatic pressure (Pdi) was flat during inspiration (despite Edi was still present to control the ventilator) [15].

In simpler words, the amount of volume supplementation by the ventilator affects the force generating capability of the diaphragm, for the same velocity of activation/shortening. The amount of unloading due to this “load subtraction” for a maintained neural effort is proportional to the amount of volume supplementation on a square-law based function.

Evidence of mechanical ventilation affecting diaphragm shortening can be found in studies using sonomicrometers implanted directly into the muscle. It has been shown in both animals and patients that passive mechanical ventilation causes shortening of the diaphragm [9, 16]. During spontaneous breathing alone without a ventilator), diaphragm length was shown to be proportional to tidal volume, and shortening velocity was proportional to inspiratory flow [9]. Edi was also found to be proportional to diaphragm shortening (and tidal volume). Other experimental evidence during submaximal breathing describes relatively linear relationships for neural activation and respiratory muscle shortening [17, 18] as well as for diaphragm velocity of shortening and inspiratory flow [17]. Taken together, it can be assumed that the diaphragm force generation will be affected by flow delivery of the ventilator, not by weakening of the muscle, but rather decreasing the load against which the diaphragm must contract.

The relationship between Edi and *P*_ES_ (at peak Edi) is linear (Fig. 5) during the LAM breaths, implying that with increasing assist, the reduction in *P*_ES_ is directly related to the reduction in Edi (deactivation causes less force production). During the assisted breaths, the esophageal pressure generation was less for a given (unchanged) Edi, suggesting that uncoupling occurred. The fact that *P*_ES_ is uncoupled means that the volume that the patient generates on the ventilator is also uncoupled. We found that the volume that can be generated (for a given Edi) during the LAM, can no longer be generated during assist (for the same Edi) because of this uncoupling.

### Critique of the study

We are confident with our results because our data were produced from 1792 comparisons to LAM breaths (including two modes of ventilation, 7 levels of assist each (2 LAM breaths per level), with 4 loads), where 80% are within plus/minus 1–2 cm H2O, when comparing *P*_L___LAM_ to *P*_L___CMV_. This is one of the strengths of the current study, that we matched the assisted breaths (during two synchronized modes) to the passive ventilation (in terms of flow and volume, *r* = 0.99), allowing us to rely on the equation of motion. We assumed that *P*_L___CMV_ is the “true” lung-distending pressure, which *P*_L___LAM_ nearly mirrored (Fig. 3, panel A).

However, several points should be considered when interpreting our data:

All analysis (quantification of variables) was performed from start of Edi to peak Edi (except for swings in *P*_ES_), which is the relevant inspiratory time, and is the period of time that Edi-controlled assist is delivered during inspiration. We chose to analyze the waveforms up to the peak of Edi because this is the point of maximal pressure delivery (both NAVA and NPS), and after the peak Edi, the muscles start to relax, and any increase in pressure would be due to recoil of the respiratory system. It would be more difficult to determine the contribution to *P*_L_ (between a patient’s effort in relation to the ventilator pressure delivery) if asynchrony were present. The only exception in our analysis was for the driving transpulmonary pressure which, because of the reversal of *P*_ES_ at high assist, was calculated from start of Edi to the nadir of *P*_ES_.

In the present study, the Servo-I ventilator provided 2 cm H2O during the LAM (for both NAVA level setting of “zero”, and NPS setting of “zero”), which we corrected for in two manners: during CMV, we applied 2 cm H2O in PC mode, to obtain the volume generated and applied this “volume correction”. During NAVA and NPS, we corrected *P*_L___LAM_ by subtracting the 2 cm H2O from *P*_VENT_, “pressure correction”. Comparing the two correction methods showed no difference (Fig. 4) in *P*_L___LAM_, suggesting that when this method is to be applied in patients, a pressure correction of *P*_L___LAM_ would be sufficient, and no passive ventilation (NM blockade) would be required.

Admittedly, in the current study, we did not repeat the evaluations of *P*_L___LAM_ at different levels of PEEP. The main reason for this is the potentially long duration of the protocol (if we were to repeat the study at different PEEP levels). Applying PEEP in “lung-healthy” rabbits, would most likely cause problems with hemodynamics and would increase the risk of early termination of the study, which would not be helpful since we needed the period of CMV and paralysis at the end to compare our P_L_ predictions. Of course, it will be necessary to perform a study in lung-injured rabbits, and to change the PEEP. We are, however, encouraged that our results would not be affected by PEEP because: (i) All parameters were taken above PEEP, as the “swing” or “inspiratory” portion; (ii) we had the same PEEP for CMV, LAM, and assisted modes, (iii) banding—which affects the respiratory mechanics, see Figure E3 in Additional file 4—did not influence the estimate of *P*_L__*P*_ES_, nor *P*_L___CMV_, by the *P*_L___LAM_.

Lastly, the results of our study need to be confirmed in patients undergoing mechanical ventilation for respiratory failure. We are encouraged by the clinical study of Bellani and co-workers who found a good correlation between *P*_L__*P*_ES_ and *P*_L___CMV_ when flows and volumes could be matched during the two conditions [19].

## Conclusion

In spontaneously breathing rabbits on synchronized mechanical ventilation, we were able to show that a single LAM breath allows for predictions of Δ*P*_L_, “*P*_L___LAM_”, without measuring esophageal pressure, and follows a mathematical transfer function, based on a square law, that explains how unloading occurs. We demonstrated in animals with elastic or resistive loads, that increasing assist with either NAVA or NPS, unloads the breathing by a combined action of volume supplementation and load subtraction.

### Supplementary Information

**Additional file 1.** Supplementary text provided for derivation of simpler PL_LAM prediction.

**Additional file 2.** Figure with examples of measured waveforms with increasing NPS.

**Additional file 3.** Figure with Pα included to demonstrate it's overestimation.

**Additional file 4.** Figure demonstrating the effect of banding on inspiratory holds (NAVA and NPS).

## Data Availability

The datasets used and/or analyzed during the current study are available from the corresponding author on reasonable request.

## Abbreviations

LAM: Lower assist maneuver (single and acute maneuver)
Δ*P*_L_: Transpulmonary driving pressure
NAVA: Neurally adjusted ventilatory assist
NPS: Neurally triggered and cycled pressure support
VC: Volume control
PC: Pressure control
Edi: Electrical activity of the diaphragm
*P*_ES_: Esophageal pressure
*P*_L__*P*_ES_: Transpulmonary pressure calculated from esophageal pressure swings during spontaneous breathing modes
CMV: Controlled mechanical ventilation
*P*_L___CMV_: Transpulmonary pressure during controlled mechanical ventilation
*P*_L___LAM_: Transpulmonary driving pressure predicted from LAM maneuver
*P*_RS_: Pressure of the total respiratory system
*P*_CW_: Chest wall recoil pressure
*P*_L_: Transpulmonary pressure
PVBC: Patient-Ventilator Breath Contribution Index
*P*_VENT_: Ventilator pressure
*P*_GA_: Gastric pressure
PEEP: Positive end-expiratory pressure
BP: Blood pressure
HR: Heart rate
SAT: Oxygen saturation
Pα: Estimate of *P*_L_ during LAM without PVBC correction
