# Supplementary material for: Estimation of transpulmonary driving pressure during synchronized mechanical ventilation using a single lower assist maneuver (LAM) in rabbits: a comparison to measurements made with an esophageal balloon
Source: Crit Care. 2023 Aug 25;27:325. doi: 10.1186/s13054-023-04607-2 (PMC10463600; doi:10.1186/s13054-023-04607-2)
Supplement: Supplementary file 1 — Additional file 1. Supplementary text provided for derivation of simpler PL_LAM prediction. [file 13054_2023_4607_MOESM1_ESM.docx]

**ELECTRONIC SUPPLEMENTARY MATERIAL**

**ESTIMATION OF TRANSPULMONARY DRIVING PRESSURE DURING SYNCHRONIZED MECHANICAL VENTILATION USING A SINGLE LOWER ASSIST MANEUVER (LAM) IN RABBITS: A COMPARISON TO MEASUREMENTS MADE WITH AN ESOPHAGEAL BALLOON**

Ling Liu^1^, Hong-Liang Li^2^, Cong Lu^3,4,^ , Purab Patel^4^, Danqiong Wang ^5^, Jennifer Beck* ^4,6,7^, Christer Sinderby^4,7,8^

^1^Jiangsu Provincial Key Laboratory of Critical Care Medicine, Department of Critical Care Medicine, Zhongda Hospital, School of Medicine, Southeast University, Nanjing, 210009, China.

^2^Department of Critical Care Medicine, Beijing Tiantan Hospital, Capital Medical University, Beijing, China

^3^Institute of Medical Science, University of Toronto, Toronto, Canada

^4^Keenan Research Centre for Biomedical Science of St. Michael’s Hospital; Department of Critical Care, St. Michael's Hospital, Toronto, Canada

^5^Department of Critical Care Medicine, The Quzhou Affiliated Hospital of Wenzhou Medical University, Quzhou People’s Hospital, 324000, Quzhou, China

^6^Department of Pediatrics, University of Toronto, Toronto, Canada

^7^Member, Institute for Biomedical Engineering and Science Technology (iBEST) at Ryerson University and St-Michael’s Hospital, Toronto, Canada

^8^Department of Medicine and Interdepartmental Division of Critical Care Medicine, University of Toronto, Toronto, Canada

*Corresponding author: Jennifer Beck, Keenan Research Centre for Biomedical Science of St. Michael’s Hospital; Department of Critical Care, St. Michael's Hospital, 30 Bond Street, Toronto, Ontario, Canada, M5B1W8; Telephone: 416-880-3664 Email: [jennifer.beck@rogers.com](mailto:jennifer.beck@rogers.com)

**METHODS**

Calculation of Pα:

Pα is an independent calculation and estimate of P_L_ (under the same principles of P_L___LAM_) but not corrected for the PVBC.

STEP 1: LAM maneuver to get LAM volume

STEP 2: Calculate Ventilator Volume (VOL_VENT_)= Tidal volume – LAM volume

STEP 3: Calculate load factor α = P_VENT_/VOL_VENT_

STEP 4: Calculate Pα = Tidal volume x load factor α

*Note this is different from P_L___LAM_ because there is no correction of the LAM volume for PVBC.

**RESULTS**

Figure E1 illustrates measured and predicted waveforms of variables with increasing NPS levels (left to right 4-16 cm H2O) for single breaths during assist (green = assisted breaths) and during the LAM (red waveforms, LAM breaths). Where appropriate, signals for PC mode (passive ventilation) are included (dark blue waveforms). The waveforms are presented for one representative subject with high resistive load applied. The first peak of Edi (whether occurring for assisted or LAM), is indicated by the vertical royal blue bar; Cycling-off time point of NPS indicated by the vertical green bar).

In this example, increasing the assist from 4 to 8 cmH2O increased most variables including, ventilator flow, tidal volume, P_L__P_ES_ (yellow) and P_L___LAM_ (orange). This initial 4 to 8 cm H2O in NPS did not really alter the variables during the LAM. This response (in this particular subject) shows that the first level of assist increases ventilator driving pressure, flow and volume, without a reduction in neural or mechanical inspiratory effort. However, further increasing NPS to 12 and 16 cmH2O further increased flow, volume, P_L__P_ES_ and P_L___LAM_, however at these levels of assist, the corresponding values for the LAM decreased, showing that at higher levels of assist, increasing flow and volume were also associated with reduced neural and/or mechanical inspiratory efforts.

Important to note, increasing NPS shifted the minimum (nadir) of the P_ES_ waveform for the assisted breath (green dashed waveform) earlier in time, towards the onset of inspiration, and reversed the P_ES_ waveform towards more positive values. As the NPS levels increased, ΔP_ES_ during assist becomes increasingly dissociated from ΔP_ES_ during the LAM, despite the same Edi. This also means that the calculated P_L__P_ES_ waveform (yellow) starts to decrease after the P_ES_ waveform reaches its nadir, despite that volume is still increasing.

As depicted in the upper panels, P_L___LAM_ showed larger oscillations than P_L___CMV_ (blue) and P_L__P_ES_ (yellow) during early parts of the breath and could also demonstrate a large overshoot after the peak of Edi was reached. Despite this, a close fit was found between P_L___LAM_ (orange) and P_L___CMV_ (blue) at the peak of Edi. This is different for P_L__P_ES_ (yellow) which could reach its peak before the peak Edi (royal blue vertical line) and could often decrease with continuing inspiration.

Figure E2 demonstrates the group mean data for measured and predicted pressures, similar to Figure 2 in the main manuscript, however we include Pα in this Figure.

The predicted pressure Pα (open orange circles, orange solid line) over-estimated all measured pressures obtained during paralysis, including P_L___CMV_, and P_RS_ obtained during CMV, and did not consistently follow the increasing assist levels. Pα actually overestimated P_L__P_ES_ by 10 cm H2O on average.

The predicted pressure P_L___LAM_ (closed orange circles, orange line), was closely similar to P_L___CMV_ for resistive loads during both NAVA and NPS. During unbanded conditions, P_L___LAM_ was also similar to P_RS_, and slightly higher than P_L___CMV_ (by a few cm H2O), but this phenomenon changed when banding was applied. Banding increased P_RS_ obtained during CMV (black dashed line). With banding, P_L___LAM_ was similar to P_L___CMV_ and was far below the measured P_RS_.

Figure E3 demonstrates the impact of banding on the relationship between ventilator pressure and volume during the end-inspiratory holds. During both NAVA (Panel A) and NPS (Panel B), banding caused a change (increase) in the pressure required to reach the hold volume (as noted by the shift of the curve to the right from unbanded, orange symbols to banded, blue symbols).

**DISCUSSION**

**Mathematical derivation of P_L___LAM_**.

Original P_L___LAM_ equation provided in the Methods:

Known variables at the start:

1. **LAM volume** (i.e. patient-generated volume alone, during a single breath where the assist is reduced to 0 cm H2O in NPS, and 0 cm H2O/uV during NAVA.
2. **Tidal volume** (total volume delivered and includes both patient and ventilator) during NPS or NAVA
3. **Ventilator pressure** during NPS or NAVA

Need to solve:

**Ventilator volume** (VOL_VENT_) alone.

This would allow us to obtain the global load the ventilator (or the patient, or both) needs to overcome, so need to calculate P_VENT_/VOL_VENT_.

1. Ventilator Volume (VOL_VENT_) = Tidal volume – (LAM volume * PVBC),

where PVBC = (LAM volume/Tidal volume)

1. Calculate **load factor**: P_VENT_ /VOL_VENT_
2. Calculate **P_L___LAM_** = Tidal volume * load factor

P_L___LAM_ is the driving pressure that BOTH patient and ventilator need to generate to overcome the global load.

Equation 3, re-written:

1. P_L___LAM_ = Tidal volume * (P_VENT_ /VOL_VENT_)

^#^note P_VENT_ is a “delta” or “difference” in pressure: assist pressure minus LAM pressure, which in this protocol, was always 2 cm H2O, so ΔP_VENT_

1. P_L___LAM_ = (Tidal volume x ΔP_VENT_)/(Tidal volume –[LAM volume * PVBC])
2. P_L___LAM_ = (Tidal volume x ΔPvent)/(Tidal volume –[LAM volume * (LAM volume/tidal volume)])

To simplify, divide top and bottom by tidal volume:

1. P_L___LAM_ = ΔP_VENT_ / (1-(LAM volume/tidal volume)^2^)

Where ΔP_VENT_ is the difference in ventilator-delivered pressure for the assisted and the LAM breaths.

1. **P_L___LAM_ = ΔP_VENT_ / (1- PVBC^2^)**

So P_L___LAM_ becomes a ratio of the difference in ventilator pressure between two breaths (numerator) corrected for the PVBC (denominator), i.e. the relative contribution of patient and ventilator.

This is a simpler equation since each variable appears only once.

**FIGURE LEGENDS**

Figure E1: Sample waveforms during increasing levels of assist with Neurally-Triggered and Cycled Pressure Support (NPS)

Top panel shows tracings of P_VENT_ (green), P_L_CMV_ (dark blue), P_L_PES_ (yellow) and P_L___LAM_ (orange). Second panels from the top show tracings of P_ES_ and Edi with assist (green) and during the LAM (red). Third from the top panels show single-breath tracings for flow during PC (blue), NPS (green) and during the LAM (red). Bottom panels show the corresponding volumes (PC, blue; NPS, green; LAM, red). The blue vertical solid lines indicate the “first” peak of Edi (for matched Edi NPS vs. LAM, whichever comes first), and the green vertical line indicates when the NPS cycled off (based on flow). The black dashed vertical lines show the point of most negative P_ES_ (downward arrow i.e. the nadir) and the peak P_L__P_ES_ (upward arrow pointing to yellow curve). The 1-PVBC^2^ values (i.e. indicating the contribution of the ventilator to the breath) increased from NPS=4 (1-PVBC^2^ = 0.25) to NPS=8 (1-PVBC^2^ = 0.41) to NPS=12 (1-PVBC^2^ = 0.66) to NPS=16 (1-PVBC^2^ = 0.83).

Figure E2: Group mean data for the measured and predicted pressures during all conditions

Measured and predicted pressures, as well as diaphragm activity (y axis) are provided for increasing assist (x axis) with NAVA (left panels), and NPS (right panels), as indicated. The results for resistive loads (Low and High) PANEL A, and unbanded and banded PANEL B conditions are presented. P_VENT_ is displayed as the pale grey solid shading; P_RS_, blue empty circles and blue dashed lines; Pα = orange, open circles; P_L___LAM_ = orange, solid circles; P_L___CMV_ blue solid circles blue solid line; P_L__P_ES_, yellow solid circles and solid line; Note that Edi assisted (green circles) and Edi LAM (red circles) are nearly identical.

Figure E3: Pressure-volume relationship for inspiratory holds with banding

X axis: end-inspiratory hold pressure; y axis: volume at end inspiratory hold. Orange symbols: unbanded condition. Blue symbols, banded condition. Panels A and B are results for inspiratory holds performed during NAVA and NPS, respectively. The banding caused a shift (increase) in the hold pressure for a given hold volume.
