# Supplementary figures and images for: Estimation of transpulmonary driving pressure during synchronized mechanical ventilation using a single lower assist maneuver (LAM) in rabbits: a comparison to measurements made with an esophageal balloon
Source: Crit Care. 2023 Aug 25;27:325. doi: 10.1186/s13054-023-04607-2 (PMC10463600; doi:10.1186/s13054-023-04607-2)

## Slide 1
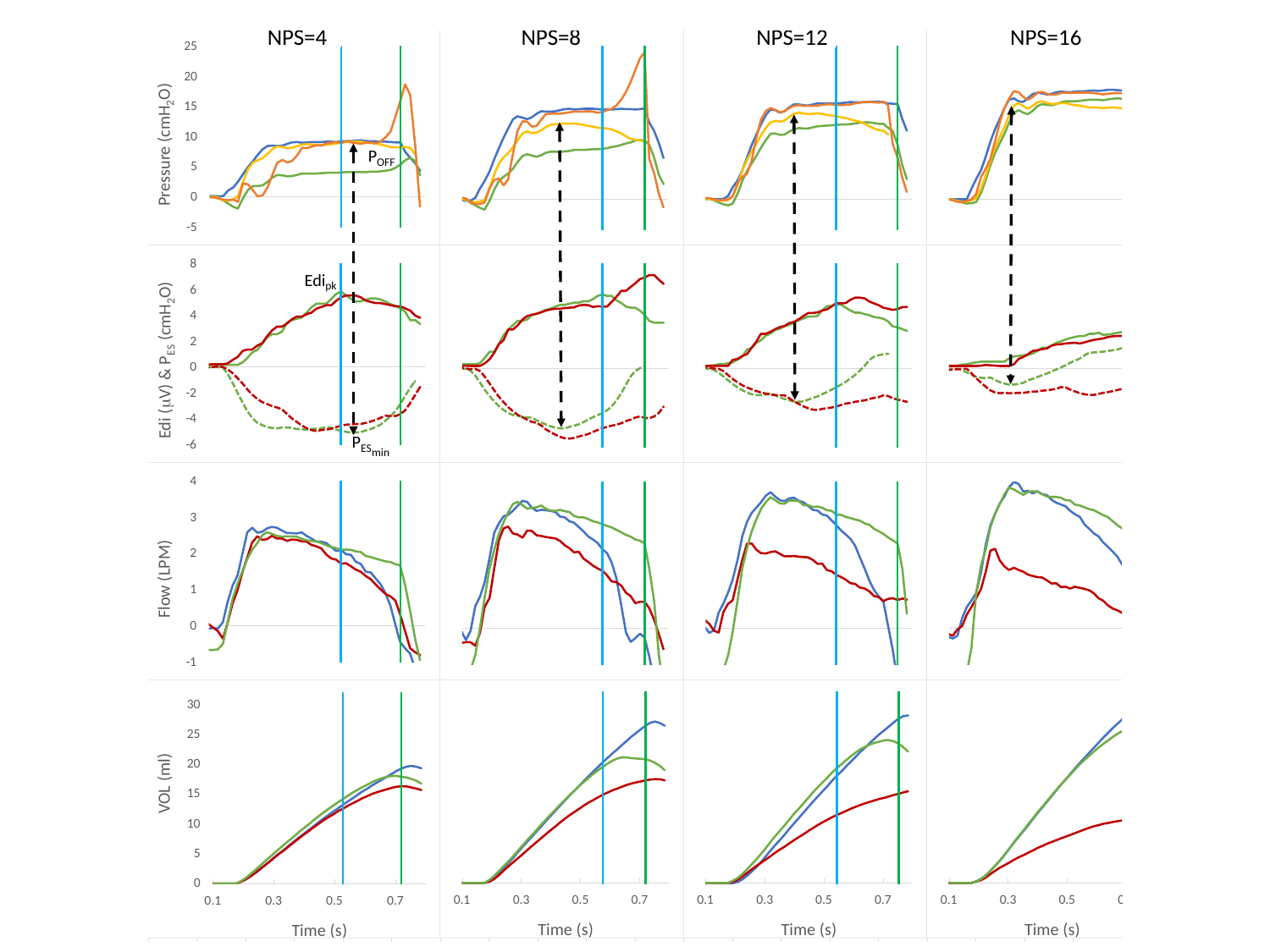

Supplement: Supplementary file 2 — Additional file 2. Figure with examples of measured waveforms with increasing NPS. [file 13054_2023_4607_MOESM2_ESM.pptx]

## Slide 1
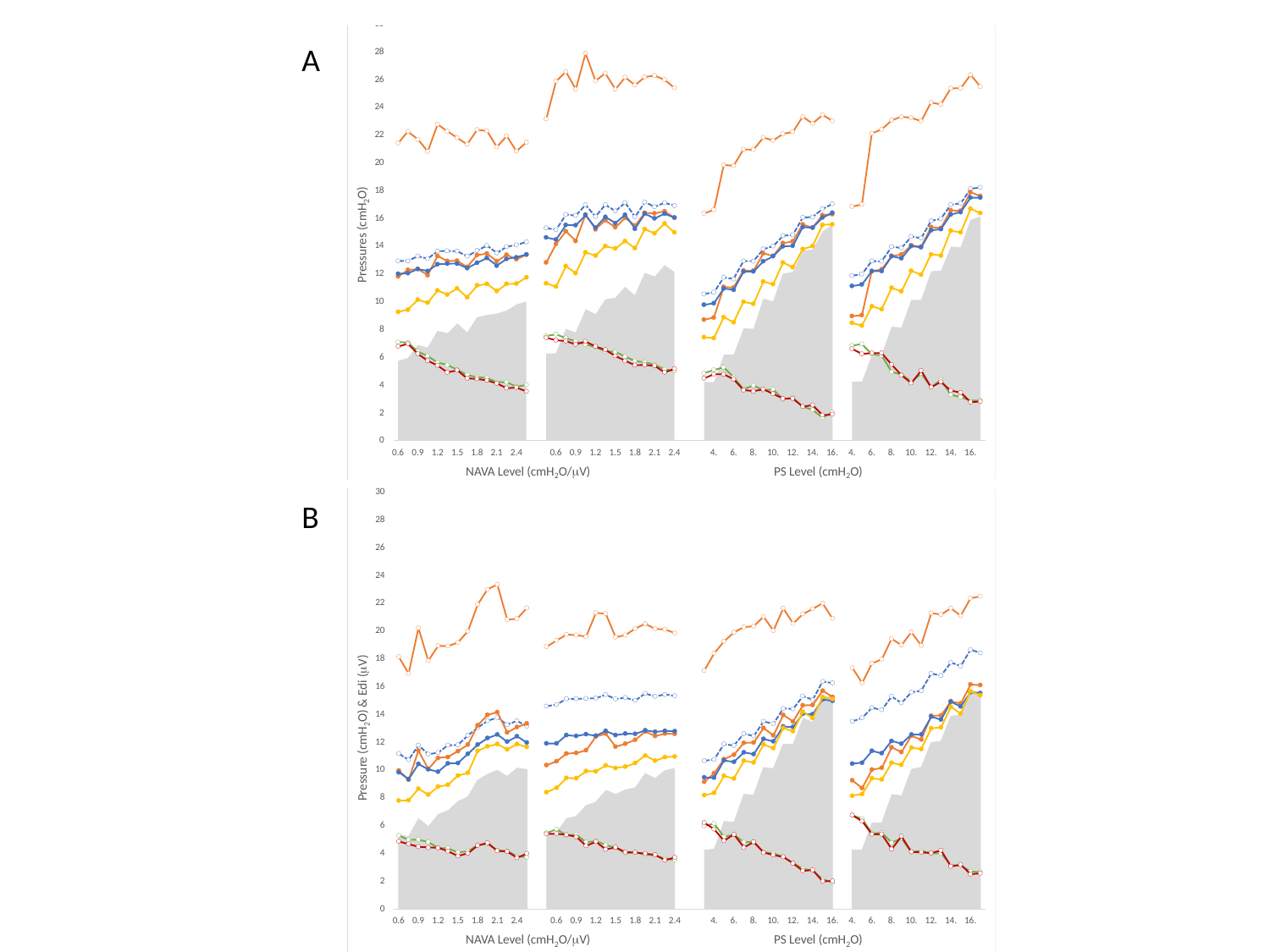

A
B

Supplement: Supplementary file 3 — Additional file 3. Figure with Pα included to demonstrate it's overestimation. [file 13054_2023_4607_MOESM3_ESM.pptx]

## Slide 1
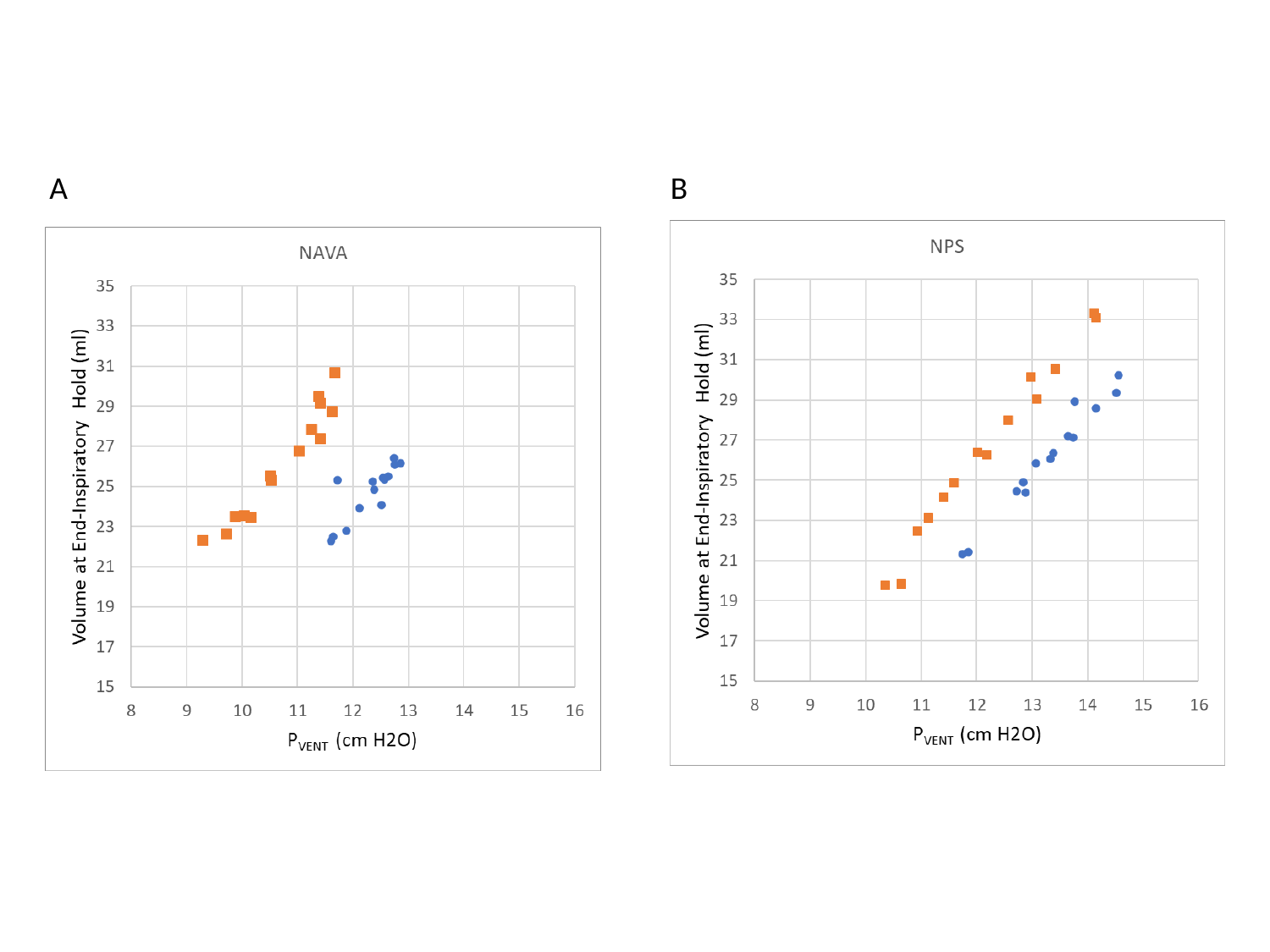

A B

Supplement: Supplementary file 4 — Additional file 4. Figure demonstrating the effect of banding on inspiratory holds (NAVA and NPS). [file 13054_2023_4607_MOESM4_ESM.pptx]
